# Temporal evolution of postsurgical bone repair in a rabbit model: A [99mTc]Tc-MDP scintigraphic study

**DOI:** 10.1590/1414-431X2024e12953

**Published:** 2024-03-18

**Authors:** A. Yoneda, K.J.C.C. de Lacerda, L. Alexandre-Santos, E.N. Itikawa, P. Louzada-Junior, L. Wichert-Ana

**Affiliations:** 1 Laboratório de Medicina Nuclear e PET/CT, Departamento de Imagem Médica, Hematologia e Oncologia Clínica, Hospital das Clínicas, Faculdade de Medicina de Ribeirão Preto, Universidade de São Paulo, Ribeirão Preto, SP, Brasil; 2 Divisão de Reumatologia, Departamento de Medicina Interna, Faculdade de Medicina de Ribeirão Preto, Universidade de São Paulo, Ribeirão Preto, SP, Brasil; 3 Instituto de Física, Universidade Federal de Goiás, Goiânia, GO, Brasil

**Keywords:** Bone scan, Rabbits, Experimental study, Osteotomy, Bone regeneration, Nuclear medicine

## Abstract

Bone regeneration is crucial for repairing bone tissue following various injuries. Research techniques that enable the study of metabolic changes in bone tissue under different conditions are important for understanding bone repair and remodeling. This study used bone scintigraphy to evaluate osteogenesis secondary to osteotomy in a preclinical model of New Zealand rabbits. For this purpose, we conducted a longitudinal, prospective, case-control study in which scintigraphic variables were measured in both the right forearm (case-operated) and the left forearm (control - non-operated). The study sample consisted of 10 rabbits subjected to osteotomy, followed by a 12-week postoperative evaluation period, divided into six imaging stages at 1, 2, 3, 4, 8, and 12 weeks. We observed that the operated forearm showed significantly higher external radiation than the control side, using the pinhole collimator, denoting an increase in the biodistribution and tropism of the radiopharmaceutical to the operated forearm. Among the three evaluated time points, osteoblastic activity was highest in the second week and presented a significant decline in the 8th and 12th weeks, denoting regeneration and resolution of the surgical injury; the control forearm was also influenced by the inactivity imposed by the operated forearm. This fact was notably evidenced by the reduction in the metabolic activity of osteoblasts in the left forearm. Our study suggested that bone scintigraphy was sensitive enough to semi-quantitatively differentiate the metabolic activity of osteoblasts in the operated forearm in the three temporal landmarks evaluated in the study.

## Introduction

The human skeleton has a variety of functions that go far beyond supporting the body. Bones provide structural support for the rest of the body, allow circulation and movement through levers for muscles, protect vital internal organs and structures, maintain mineral homeostasis and acid-base balance, serve as a reservoir of growth factors and cytokines, and provide the ideal environment for hematopoiesis by the bone marrow (1). Bone is a dynamic material constantly exposed to mechanical influences that challenge its structural integrity. There are various causes of bone fractures, but unlike inert materials, bone can regenerate and form new tissue where it has been damaged.

Various types of accidents can result in severe fractures with loss of bone segments, which concerns various parts of society and health researchers, as there are still no efficient protocols for treating certain types of fractures with reconstruction and repair of bone segment losses (2). In addition, the literature shows that up to 20% of fractures evolve with a delayed or no repair. Among the factors contributing to this complication of non-consolidation is the location of the bone lesion, the treatment method applied, and the characteristics of the patient's lifestyle and type (3,4).

Different methodologies exist for studying cellular metabolic activity and bone repair in bone defects. A simple radiology examination is one of the methods used for evaluating bone repair and can be used to assess the evolution of bone defect repairs in the skeleton (5,6). However, there are other methods, such as nuclear magnetic resonance (7), computed tomography (8), ultrasound (3), and bone scintigraphy (3,9). Bone scintigraphy is an imaging procedure that demonstrates bone metabolic activity. In bone scintigraphy, the radiopharmaceutical methylene diphosphonate (MDP) labeled with technetium-99m (99mTc-MDP) rapidly accumulates in the bone by adsorption in the organic mineral phase, and the image can be acquired 2 to 3 h after intravenous administration. The radionuclide study may reveal an anomaly long before the radiological alteration (5). In some situations, it may indicate which area should be more thoroughly investigated.

In other situations, however, bone scintigraphy may be valuable in demonstrating a pattern of abnormal changes that will reinforce the radiological findings. It can also indicate if the radiologically detectable lesion is clinically significant (10-12). This examination can be used in various situations, such as research, detecting changes in animal bone metabolism, and evaluating the effect of substances that would stimulate osteogenesis in bone defects (12,13).

In this study, we propose a protocol for using the bone scintigraphy technique to analyze the metabolic activity of osteoblasts in a bone defect in the foreleg of New Zealand rabbits using specific regions of interest (ROIs). This approach differs from methods that compare radiotracer uptake between the defective bone and the adjacent one. Defining ROIs increases accuracy by allowing direct comparison in specific areas of the defective bone. This is important due to variations in metabolic activity in different parts of the bone during different healing phases.

## Material and Methods

This study followed the Ethical Principles in Animal Experimentation Protocol No. 080/2008 adopted by the Brazilian College of Animal Experimentation (COBEA) and was approved by the Animal Ethics Committee (CETEA) of the Ribeirão Preto Medical School, University of São Paulo (FMRP-USP). The sample consisted of 10 male New Zealand rabbits aged 8 to 10 weeks with an average weight of 3.068±0.755 kg. All animals underwent scintigraphy examinations in the first, second, third, fourth, eighth, and twelfth postoperative weeks. They were housed in individual cages throughout the experiment, with strict cleanliness and *ad libitum* water and feed, at 25±2°C, and a 12-h light/dark cycle. All animals were euthanized following the guidelines recommended in the AVMA Guidelines on Euthanasia (Formerly Report of the AVMA Panel on Euthanasia, June 2007) (4).

### Surgical intervention

**Figure 1:**
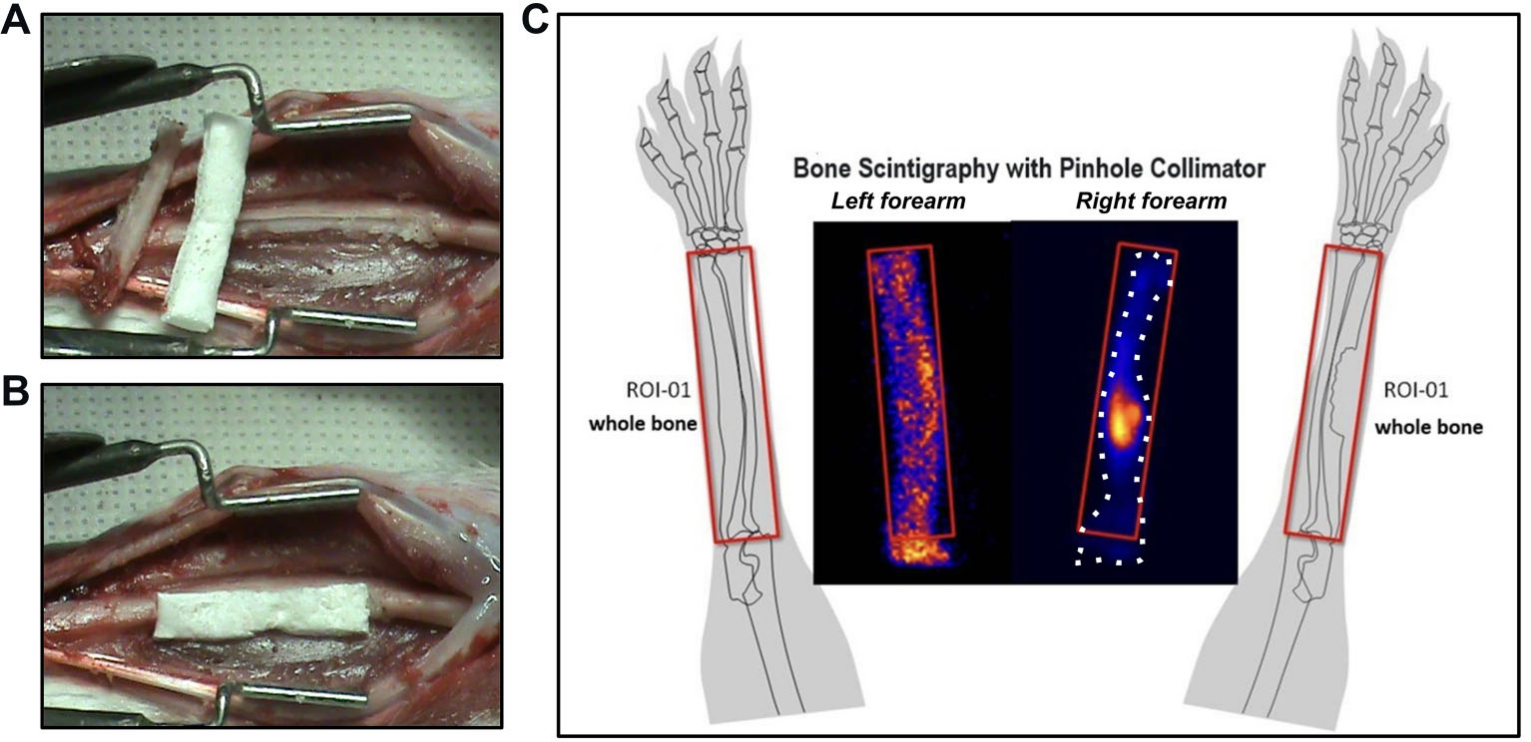
Surgical and scintigraphic aspects of the study. **A**, Preparation of the hemostatic sponge during creation of the bone defect in the rabbit. **B**, Placement of the hemostatic sponge in the bone defect. **C**, Bone scintigraphy images were obtained with a pinhole collimator of the radius and ulna of the right forearm with the bone defect and the left forearm (control). The animal's right forearm silhouette is within the white dotted line. The radiopharmaceutical's focal intense uptake in the bone defect region suppressed the visualization of the smaller uptake in the rest of the bone due to image normalization using maximum intensity projection (MIP).

Anesthesia was induced using 10% ketamine hydrochloride (Ketamine Ageneremph^®^, Brazil; 11.54 g in 100.00 mL of diluent, 0.25 mL/kg was used) and xylazine (Dopaseremph^®^; Calier, Brazil; 200 mg in 10 mL of diluent, 0.15 mL/kg was used) injected subcutaneously. A 2.0 cm incision was made at the junction of the middle third with the distal part of the right forearm to expose the ulna bone. Then, circumferential deperiostization of the entire ulnar shaft was performed. Next, osteotomy was performed by placing a metal template on the ulnar shaft as a marker for the 2.0 cm osteotomy site. The medullary canal was then blocked proximally and distally with bone wax, and the bone defect was filled with an absorbable hemostatic sponge (HA) in all animals (Figure 1).

Muscle fascia was sutured, and the skin was closed with appropriate occlusive dressings. The animals were medicated subcutaneously during the postoperative period with the following drugs: ketofen 10% (Ketoprofen 10.0 g diluted in 100.0 mL), small size multi-biotic (Vitalfarma Pharmaceutical Industry Ltd., Brazil) based on benzyl penicillin 1,200 IU/mg, procaine benzyl penicillin 995 IU/mg, potassium benzyl penicillin 1,590 IU/mg, and streptomycin base (sulfate) 9,790 mcg/mg.

### Bone scintigraphy

Bone scintigraphy was performed on the right forearm (case) and left forearm (control) of the rabbits. With the animals anesthetized, 10 mCi of the radiopharmaceutical MDP labeled with Technetium-99m (^99m^Tc) was administered through a venous puncture. The rabbits were kept at rest for 3 h to allow tracer uptake by the organic matrix of the bone tissue. MDP is a drug that emits pure gamma radiation with 140 keV energy, has a physical half-life of 6 h, has a tropism for the organic matrix of the bone, and concentrates in more significant quantities as cellular metabolic activity increases. At the time of the exam, the rabbits were anesthetized again and placed on an acrylic plate for fixed positioning of the front paws under the apex of the pinhole collimator. The forearm images were magnified on the detectors.

A static image of each forearm was acquired for 10 min using a 128×128 matrix without digital zoom, only with the physical magnification of the pinhole collimator positioned 3.0 cm from the orifice. The equipment's photopic was centered at 140 keV, and the energy window was configured to 20%. The obtained scintigraphic images can be seen in Figure 1, which were used to generate the semi-quantitative ROIs for the study, such as the one observed in ROI 01.

### Evaluation of the radiopharmaceutical biodistribution on forearms

The counting was obtained from the median region between the wrist and the elbow of each paw, in which the number of gamma rays registered in that incidence was analyzed during the 1st, 2nd, 3rd, 4th, 8th, and 12th weeks of the postoperative period.

From these images, relative semi-quantifications were performed on four study areas in the rabbit forearms, which were drawn as ROI. ROI 01 involved the entire length of the forearm bones (ulna and radius), and was divided into thirds, with ROI 02 involving the distal third, ROI 03 involving the middle third, and ROI 04 involving the proximal third. Pixel counts were performed in these ROIs on both forearms of the animal. ROI 00 was a soft tissue area, which was standardized in the forearm images.

### Statistical analysis

A mixed-effects regression model was used for data analysis. The model allowed for estimating the means of the dependent variables in each interest group and the difference between these means. In addition, 95% confidence intervals were calculated for the means, and when the interval did not include the value of zero, the difference between the groups was considered significant (analogous to P<0.05). The SAS-9.0 software (USA) was used for the analysis. The graphs were generated in the R-3.0.1 software (R Core Team).

## Results

### Results of the scintigraphy examinations

Table 1 describes the metabolic activity analysis of ROIs 00 to 04 for both the left and right forearms. As can be seen in the table, the estimated difference in scores between the two forearms was greatest in ROI 01 involving the entire length of the limb. The second highest scoring area was ROI 03, which was operated. The other ROIs 02 and 04 showed smaller differences between the two forearms. All these differences were significant and showed that bone structures and soft tissues had greater radiopharmaceutical accumulation in the operated paw.

**Table 1:** Estimated difference of the osteogenic activity analysis in counts/pixel in scintigraphic images, between the right and left forearm in each region of interest (ROI) in the hemostatic sponge group, with confidence intervals and P-values.

ROI	Estimated differenceRight - Left	95%CI		P-value
		Lower limit	Upper limit	
00	55.16	4.67	6.36	0.0001
01	86.65	82.55	90.74	0.0001
02	28.50	24.96	32.03	0.0001
03	81.20	74.72	87.67	0.0001
04	15.88	13.19	18.57	0.0001

ROI: 00: reference soft tissue; 01: entire length of the forearm bones (ulna and radius); 02: distal third of forearms; 03: middle third; and 04: proximal third or forearms.

Figure 1 illustrates the surgery performed on the right forearm. The bone defect produced shown in Figure 1A was covered with a hemostatic sponge (1B). The scintigraphic images of the two forearms (1C) showed a significant increase in osteogenesis in the projection of the middle third of the paw, corresponding to the surgical area.

**Figure 2:**
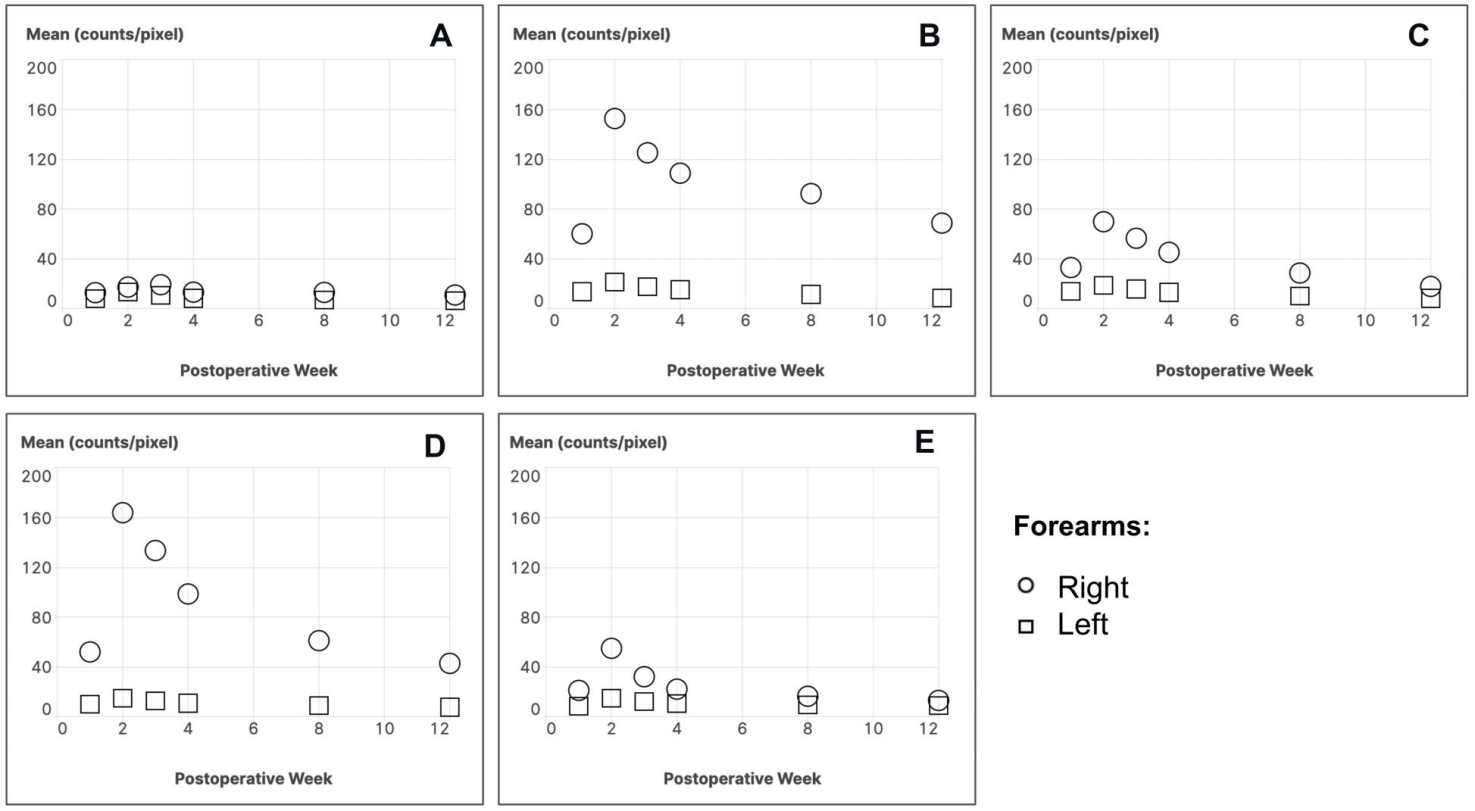
Temporal evolution of osteogenesis within the five regions of interest (ROI) in both right (operated) and left (control) rabbit forearms at 2, 4, 6, 8, 10, and 12 postoperative weeks. The data are reported as mean counts per pixel obtained from ROI drawn over both forearms. **A**, reference soft tissue, ROI 00; **B**, the entire length of the forearm bones (ulna and radius), ROI 01; **C**, distal third of forearms, ROI 02; **D**, middle third of forearms, ROI 03; and **E**, proximal third of forearms, ROI 04.

Figure 2 shows a graphical representation of the pixel count values in the five ROIs. ROI 00 visually exhibited no significant difference between the right (operated) paw and the left, but quantitatively the difference was significant. The graphs of ROI 01, 02, 03, and 04, which comparatively demonstrated the pixel counts of the left and right forearms, already showed lines with very different shapes. The right forearm had a peak in the 2nd postoperative week and decreased until the 4th postoperative week. The maximum pixel count values were detected in ROI 03.

## Discussion

In this paper, we describe a longitudinal, prospective, case-control experimental study evaluating the osteogenesis in the right and left forearms of rabbits using scintigraphy. The experiment lasted for a 12-week postoperative evaluation period. Scintigraphy is a complementary exam that can be performed to evaluate bone tissues, and its interpretation can help diagnose and anticipate the presence of other asymptomatic diseases (4,9,12). For example, in an experimental study with rabbits, bone scintigraphy allowed for the early identification of the onset of osteoarthritis (10,11).

The bone is a complex tissue, and remodeling is regulated by osteoblasts for bone formation. There are many ways to analyze and interpret the regeneration bone. The use of fluorochrome labeling, histomorphometry, and biopsy are considered invasive methods. In contrast, imaging technologies provide a non-invasive evaluation of bone anabolism. Methods frequently used for imaging are classical X-ray, peripheral computed tomography, and functional imaging with bone scintigraphy (14). Scintigraphy is an exam that can demonstrate the activity of osteoblastic cells in the region of the surgically-induced bone defect (4,8).

Osteogenesis analyses were performed every week of the postoperative period in each ROI. The values were higher in the first three weeks, and the maximum values of pixel counts were observed between the 1st and 2nd weeks, declining in the 3rd postoperative week, where the metabolic activity of osteoblasts begins to decrease. Many authors demonstrated in experimental studies that scintigraphic findings of the first and fourth postoperative weeks did not show significantly increased osteoblastic activity (15). Several studies have demonstrated that complete healing was observed in the 4th postoperative week (14,16).

The most significant difference in pixel count between the left and right forearms of the same ROI was observed in ROI 03 in the 2nd postoperative week, in which the right forearm presented a much higher average pixel count than the left forearm. Conversely, the smallest difference in pixel count between the two forearms was observed for ROI 00 of the 2nd postoperative week but with a minimal difference in pixel count between the forearms. In other similar studies, the cell turnover between bone formation and reabsorption decreases at these times of the post-operatory period, reflecting the combined effects of bone blood flow and osteoblastic activity on bone tracer kinetics (14,17-19).

It was noted that the metabolic activity of osteoblasts was low between the 8th and 12th weeks, indicating that healing had occurred in the surgical bone defect region. As expected, pixel counts were higher in the right forearm, as it suffered a bone injury, which resulted in a greater metabolic activity of the osteoblasts. The primary role of osteoblasts is to produce new bone during skeletal development and remodeling. Throughout this process, osteoblasts directly interact with other cell types within bone, including osteocytes and hematopoietic stem cells. Osteoblastic cells also indirectly signal bone-resorbing osteoclasts via the secretion of a homotrimeric protein and is typically bound to osteoblast and activated T cell membranes. Through these mechanisms, cells of the osteoblast lineage help retain the homeostatic balance between bone formation and bone resorption. The same was observed in the bone defect region, which was expected (20).

The bone scintigraphy method effectively indicated the metabolic activity of osteoblasts in the bone defect. There was a higher biodistribution of the radioisotope in the operated forearm compared to the non-operated forearm. Between each scintigraphy examination, osteoblast activity was higher in the 2nd postoperative week. We concluded that the bone scintigraphy examination technique was efficient for quantitatively evaluating the activity of osteoblasts in operated bones.

Scintigraphy is a widely used imaging method in medical practice to assess the function of internal organs and tissues in the body. This method offers several advantages and disadvantages that should be considered when choosing the most appropriate imaging technique for a particular patient or clinical situation. One of its main advantages is its high sensitivity in detecting functional abnormalities. In contrast to other purely anatomical imaging methods such as radiography, scintigraphy provides information about the functioning of organs and tissues, allowing the identification of metabolic and functional disorders. Additionally, scintigraphy uses relatively low amounts of radiation, making it safe for most patients. This aspect is important in long-term medical applications, such as monitoring thyroid function. It is a versatile tool, with applications ranging from assessment of cardiac problems, such as myocardial perfusion, to detection of cancer and bone diseases. Its ability to provide functional information is invaluable in many clinical cases.

However, this technique has some disadvantages. One of them is that more anatomical detail is required compared to techniques such as computed tomography (CT) and magnetic resonance imaging (MRI). The spatial accuracy of scintigraphy is not optimal, which can make it difficult to detect small lesions or detailed anatomical structures. Another disadvantage is the dependence on radioactive isotopes. Scintigraphy requires the administration of radiopharmaceuticals, which are radioactive materials. Although the amount of radiation is generally considered safe, proper disposal of radioactive waste is essential for the safety of patients and medical personnel. In addition, scintigraphy tends to be more time-consuming than other imaging techniques, such as conventional radiography. The time required to perform the procedure can be a limiting factor in clinical situations where speed is essential.

Various conditions can influence the effectiveness of scintigraphic methods, including pharmacological, technical, or pathological. It is essential to keep these factors in mind when planning and interpreting scintigraphy exams so that accurate and reliable results can be obtained. Concerning pharmacological influences, it is necessary to consider that certain medications may interfere with the scintigraphic process. For example, drugs that contain iodine or selenium may affect the uptake of radiopharmaceuticals by the thyroid, compromising the accuracy of the exam. Therefore, it is crucial that all medications being taken by the patient are reported to the physician before the procedure. Renal insufficiency should also be considered since patients with this condition may have difficulty eliminating radiopharmaceuticals from the body. This difficulty can result in an excessive and prolonged accumulation of radioactivity, which can affect the quality of scintigraphic images. Adequate hydration of the patient is essential for the proper elimination of the radiopharmaceutical and the reduction of background radiation. Insufficient fluid intake before the exam can impair the effectiveness of the scintigraphic procedure.

Technical errors in the administration of the radiopharmaceutical, during equipment calibration, or in the interpretation of the images can also compromise the quality of results. Therefore, the proper performance of the procedure is of utmost importance. In addition, other radioactive materials not related to the exam can interfere with the images, generating noise or artifacts. Moreover, movement during the exam can result in blurred or inaccurate images, highlighting the need for patient cooperation.

Specific medical conditions, such as local inflammation, bleeding, or infection, can also alter the radiopharmaceutical distribution in the body and affect the interpretation of results. In addition, particular physiological conditions, such as pregnancy or breastfeeding, may require special precautions due to the potential risks to the fetus or infant.
